# Perceptual training of audiovisual simultaneity judgments generalizes across spatial locations

**DOI:** 10.1177/03010066251342010

**Published:** 2025-05-21

**Authors:** Patrick Bruns, Theresa Paumen, Brigitte Röder

**Affiliations:** Biological Psychology and Neuropsychology, 14915 University of Hamburg, Hamburg, Germany; Biological Psychology and Neuropsychology, 14915 University of Hamburg, Hamburg, Germany; Department of Psychiatry and Psychotherapy, 37734 University Medical Center Hamburg-Eppendorf, Hamburg, Germany; Biological Psychology and Neuropsychology, 14915 University of Hamburg, Hamburg, Germany; 28592 LV Prasad Eye Institute, Hyderabad, India

**Keywords:** audiovisual, multisensory processing, perceptual learning, simultaneity judgment, spatial specificity, temporal binding window

## Abstract

Multisensory processing critically depends on the perceived timing of stimuli in the different sensory modalities. Crossmodal stimuli that fall within rather than outside an individual temporal binding window (TBW) are more likely to be bound into a multisensory percept. A number of studies have shown that a short perceptual training in which participants receive feedback on their responses in an audiovisual simultaneity judgment (SJ) task can substantially decrease the size of the TBW and hence increase crossmodal temporal acuity. Here we tested whether multisensory perceptual learning in the SJ task is specific for the spatial locations at which the audiovisual stimuli are presented during training. Participants received feedback about the correctness of their SJ responses for audiovisual stimuli which were presented in one hemifield only. The TBW was assessed separately for audiovisual stimuli in each hemifield before and one day after the training. In line with previous findings, the size of the TBW was significantly reduced after the training phase. Importantly, an equally strong reduction of TBW size was observed in both the trained and the untrained hemifield. Thus, multisensory temporal learning completely generalized to the untrained hemifield, suggesting that the improvement in crossmodal temporal acuity was mediated by higher, location-invariant processing stages. These findings have implications for the design of multisensory training protocols in applied settings such as clinical interventions by showing that training at multiple spatial locations might not be necessary to achieve robust improvements in crossmodal temporal acuity.

## Introduction

The often high and persistent specificity of perceptual learning for the trained location has long intrigued scientists (Fahle, 2005; Sagi, 2011; Seitz, 2017; Watanabe & Sasaki, 2015). For example, improvements in the ability to discriminate a unimodal visual stimulus feature such as orientation or motion direction after training are often abolished if the location of the stimuli in the visual field differs even slightly from the location during training, suggesting plasticity in early stages of the visual processing hierarchy (Crist et al., 1997; Karni & Sagi, 1991; Watanabe et al., 2002). Similarly, some studies found improvements in tactile discrimination performance to be restricted to the trained digit and hand (Godde et al., 2000; Harris et al., 2001). However, in some cases transfer of unimodal perceptual learning to untrained locations has been observed (Frank, 2025; Imai et al., 2003; Watanabe & Sasaki, 2015; Xiao et al., 2008). Moreover, it is unclear how these findings would translate to crossmodal perceptual learning.

Crossmodal learning has been extensively studied by passively exposing participants to audiovisual stimuli with a consistent spatial or temporal discrepancy (Chen & Vroomen, 2013). This typically results in a recalibration of the perceived location of auditory stimuli following the exposure phase, known as the spatial ventriloquism aftereffect (Radeau & Bertelson, 1974; Recanzone, 1998), or in a shift of the point of subjective simultaneity (PSS) between an auditory and a visual stimulus, known as the temporal ventriloquism aftereffect (Fujisaki et al., 2004; Vroomen et al., 2004). Studies of the spatial ventriloquism aftereffect have consistently found that recalibration is strongest for stimuli in the trained region of space and does not generalize across hemifields (Bertelson et al., 2006; Bruns & Röder, 2019b; Kopčo et al., 2009). Location-specificity has also been reported for temporal recalibration: If competing audiovisual exposure stimuli were presented in different hemifields, location-specific shifts in PSS were observed (Heron et al., 2012; Yarrow et al., 2011; Yuan et al., 2012), suggesting involvement of early sensory processing stages (Kösem et al., 2014) which may incorporate both spatial and temporal information across modalities.

Temporal order judgments and simultaneity judgments (SJs) have indeed been found to reflect the integration of spatiotemporal information (Badde et al., 2018; Lewald & Guski, 2004; Stevenson et al., 2012; Zampini et al., 2005). For example, audiovisual stimuli were less likely perceived as simultaneous when they were presented from different rather than from the same location (Zampini et al., 2005), or from central compared to peripheral locations (Stevenson et al., 2012). Similarly, audiovisual localization judgments were influenced by the temporal asynchrony of the stimuli (Slutsky & Recanzone, 2001). Moreover, perceptual learning of task-irrelevant visual motion stimuli was found to be restricted to motion directions which were temporally paired with task-relevant sounds as well as to visual field locations that spatially overlapped with the sound source (Beer & Watanabe, 2009). These findings suggest that spatiotemporal information is typically not processed separately but rather jointly determines learning outcomes in audiovisual tasks. In conflict with a location-specific recalibration mechanism, however, some studies reported generalization of temporal recalibration when the location of the audiovisual stimuli differed between exposure and test phase (Keetels & Vroomen, 2007) or the audiovisual stimuli were presented with a large spatial discrepancy (Navarra et al., 2013).

Crossmodal recalibration is known to emerge at two distinct time scales reflecting immediate adjustments on a trial-by-trial basis versus adjustments following cumulative evidence for a consistent crossmodal mismatch (Bruns & Röder, 2015; Van der Burg et al., 2015; Watson et al., 2019). Whereas immediate recalibration has been found to generalize to untrained stimuli (Bruns & Röder, 2015; Van der Burg & Goodbourn, 2015) and locations (Ju et al., 2019), cumulative recalibration is usually more specific for the trained stimulus features (Bertelson et al., 2006; Bruns & Röder, 2015; Heron et al., 2012; Recanzone, 1998; Roseboom & Arnold, 2011). Thus, it has been speculated that location-specificity of temporal recalibration emerges mainly at longer time scales (Ju et al., 2019).

Besides recalibration of the PSS induced by passively exposing participants to temporally discrepant crossmodal stimuli, the adaptivity of audiovisual temporal perception has been studied by engaging participants in perceptual training in which they received feedback about the correctness of their SJ response after each trial (Powers et al., 2009, 2012). Feedback training has been found to result in a narrowing of the temporal binding window (TBW), usually defined as the range of stimulus onset asynchronies (SOAs) which are perceived as simultaneous in at least 75% of the trials, rather than a change in PSS (Murray et al., 2016; Zhou et al., 2020). Thus, studies using feedback training might tap into different learning mechanisms than studies using passive exposure (Seitz & Dinse, 2007; Watanabe & Sasaki, 2015).

Similar to crossmodal recalibration after passive audiovisual exposure (Fujisaki et al., 2004; Vroomen et al., 2004), feedback-induced audiovisual temporal learning has been found to emerge over a single training session and does not require repeated training (Powers et al., 2009, 2012; Theves et al., 2020; Zerr et al., 2019). However, temporal PSS recalibration effects are observed immediately after audiovisual exposure, whereas feedback training-induced changes in TBW typically are not seen in a posttest immediately following the training phase on the same day but rather emerge only one day after training (Powers et al., 2009), suggesting that TBW training effects depend on (sleep-mediated) consolidation. Thus, feedback-induced audiovisual temporal learning seems to emerge at a slower time scale than crossmodal temporal recalibration, which in turn might promote learning specificity if specificity indeed increases at longer time scales (Ju et al., 2019). Consistently, previous studies have found that feedback training-induced changes in TBW are often specific for the trained stimulus material and task: For example, training effects did not generalize from simple flash-beep stimuli to speech stimuli and vice versa (De Niear et al., 2018) or from an SJ task to the temporally dependent sound-induced flash illusion in which one visual flash is typically perceived as two flashes when presented with two beep sounds (O’Brien et al., 2020; Powers et al., 2016). Thus, a transfer of learning to other tasks and stimulus types does not emerge in crossmodal temporal training involving feedback. It is, however, yet unknown whether, if the task and stimuli would be held constant, a transfer of learning to new locations would occur.

Meta-analyses have shown that neurodevelopmental and neuropsychiatric disorders such as autism and schizophrenia are associated with an atypically enlarged audiovisual TBW (Feldman, 2018; Zhou et al., 2018, 2020). It is assumed that reduced temporal precision in multisensory processing, as reflected in enlarged TBWs, results in an impaired ability to correctly integrate or separate crossmodal stimuli. As a result, clinical symptoms such as difficulties with language processing and social communication characteristic of autism spectrum disorders, or auditory hallucinations prevalent in schizophrenia spectrum disorders might arise (Wallace & Stevenson, 2014; Wallace et al., 2020; Zhou et al., 2018). Therefore, perceptual trainings of multisensory temporal functions have been considered as potentially beneficial interventions to tune audiovisual integration abilities in these clinical populations (Wallace & Stevenson, 2014; Zhou et al., 2020). However, spatial specificity of learning effects would suggest that audiovisual stimuli must be presented at multiple different spatial locations during training to enable generalized learning effects (Sürig et al., 2018), which might be difficult to implement in applied clinical settings or on mobile devices at home. Thus, apart from its theoretical implications, the question of whether transfer of audiovisual temporal learning to new locations occurs is also relevant from an applied perspective.

Here we sought to directly probe the generalization of audiovisual temporal learning to untrained spatial locations using an adapted version of the paradigm described by Powers et al. (2012) which has been identified as optimal for inducing and measuring audiovisual temporal learning effects (Powers et al., 2009; Theves et al., 2020; Zerr et al., 2019). We used a two-interval forced choice (2IFC) audiovisual SJ task to assess the TBW immediately before perceptual training (during which feedback was provided) and one day after the training. In each trial, the audiovisual stimuli were presented either to the left or to the right of fixation, allowing us to estimate the TBW separately for each hemifield. Crucially, during feedback training the audiovisual stimuli were presented only in one of the two hemifields. Thus, if feedback-induced training effects are indeed location-specific, similar to the spatial specificity reported in temporal recalibration (Heron et al., 2012), we expected a narrowing of the TBW only for stimuli presented in the trained hemifield, but not for stimuli presented in the untrained hemifield.

## Method

### Participants

Thirty-five healthy adult volunteers from the University of Hamburg were recruited for the study. The sample size of *n* = 35 was chosen to achieve (accounting for potential dropouts) at least 80% power (at a conventional α level of 0.05) to detect a directional difference between performance improvements in the trained versus untrained hemifield (corresponding to a Test×Hemifield interaction in an ANOVA design) with a medium effect size of *d* = 0.50, for which a power analysis conducted in G*Power 3.1 (Faul et al., 2009) indicated a required sample size of 27 participants. Data of two participants had to be excluded from analyses because they did not respond to the deviant stimuli at central fixation, or their SJ data could not be fitted by psychometric curves. Thus, 33 participants (18 women and 15 men) remained in the final sample. They had a mean age of 24.4 years (range 18–52 years), reported normal hearing and normal or corrected-to-normal vision, and were all except one right-handed. Participants received course credit or were compensated €7 per hour for taking part. Written informed consent was obtained from all participants prior to the study. The experimental procedure was approved by the ethics commission of the German Psychological Society (DGPs) and the study was performed in accordance with the ethical standards laid down in the Declaration of Helsinki.

### Apparatus and Stimuli

Participants were seated in a dark sound-attenuated room with their head immobilized by a chin rest. Two loudspeakers (Companion 2, Bose Corporation, Framingham, MA, USA) were placed at a distance of 50 cm with eccentricities of ±13.5° to the left and right sides of the participants’ straight-ahead position (0°). Auditory stimuli were white noise bursts which were presented at 46 dB(A) from either the left or the right loudspeaker. Two yellow LEDs, one positioned on top of each loudspeaker, were used as visual stimuli. Both auditory and visual stimuli had a duration of 15 ms. An additional multi-color LED was positioned between the two loudspeaker/LED pairs (0°) at the same height. This LED served as a central fixation point (blue) in all trials and indicated performance feedback (green or red) after trials in the training phase. Participants responded with the numeric keypad of a standard computer keyboard by pressing either “1”, “2”, or “0”. Stimulus presentation and response recording were computer-controlled via Presentation 19.0 (Neurobehavioral Systems, Berkeley, CA, USA).

### Procedure

The experimental procedure was adapted from Powers et al. (2012). All participants completed two sessions on consecutive days. The first session began with a baseline assessment of the TBW (test phase without feedback), which was followed by the training phase (with feedback). Each participant returned to the laboratory on the next day (Session 2) for a post-training assessment of the TBW (test phase without feedback) which was identical to the baseline assessment in Session 1.

#### Test Phase Without Feedback

Participants performed a 2IFC audiovisual SJ task in which they had to determine whether the first or the second of two audiovisual stimulus pairs was presented synchronously by pressing either the “1” or the “2” key, respectively. Each trial began with the illumination of the central blue fixation LED. After a random delay between 500 and 700 ms, the first audiovisual stimulus pair was presented, which was followed by the second audiovisual stimulus pair with a separation of 1000 ms. Immediately after the response, the blue fixation light was turned off for 500 ms before the next trial began. One of the two stimulus pairs in each trial was always presented synchronously, whereas the SOA of the other stimulus pair was either ±300, ±250, ±200, ±150, ±100, ±50, or 0 ms (negative values indicating auditory leading and positive values indicating visual leading). All SOAs were equiprobable, and the synchronous pair was presented equally often in the first and in the second interval. Note that the 0 ms SOA condition was included as catch trials in which there was no correct answer (both stimulus pairs were synchronous in this condition). In half of the trials each, both audiovisual stimulus pairs were presented in the left or in the right hemifield. Each of the 52 stimulus conditions (2 hemifields×2 intervals×13 SOAs) was presented 10 times, resulting in 520 trials overall which were presented in a randomized order and subdivided into four blocks of 130 trials each.

To ensure that participants kept central fixation, the central fixation LED was occasionally turned off for 15 ms in one of the two intervals at half of the SOA between the audiovisual target stimulus pair (i.e., synchronous to the audiovisual stimulus pair in the 0 ms SOA condition). This occurred in 10% of the trials (one trial per stimulus condition) overall. Participants were instructed to refrain from responding to the SJ task in these deviant trials and to press a different key (“0”) instead.

#### Training Phase With Feedback

The procedure was identical to the test phase, with the following three exceptions: First, during the training phase audiovisual stimulus pairs were exclusively presented in either the left or the right hemifield (counterbalanced across participants). Second, the range of SOAs for the asynchronous pair was restricted to ±150, ±100, and ±50 ms. Third, participants received feedback about the correctness of their response after each trial; immediately after the response, the central fixation LED turned green (indicating correct responses) or red (indicating incorrect responses) for 500 ms (i.e., until the start of the next trial). Participants completed three training blocks of 120 trials (2 intervals×6 SOAs×10 repetitions) each. As in the test phase, 10% of the trials (one per stimulus condition and block) were deviant trials in which the central fixation light flickered in one of the two intervals. Response feedback in these trials was based on whether or not participants correctly responded by pressing the “0” key.

### Data Analysis

All performance measures were calculated separately for each test phase (pretest versus posttest) and hemifield (trained versus untrained). First, deviant trials (flickering of the central fixation LED) and catch trials (0 ms SOA in both intervals) were extracted and analyzed separately from the main trials. For deviant trials, the hit rate (i.e., the percentage of trials in which the “0” key was pressed) was calculated for each participant to verify that participants indeed kept fixation during the test phases. Catch trials (for which there was no correct response) were used to determine a potential bias to respond “1” or “2” by simply averaging responses for each participant in this condition. Finally, false alarms (i.e., “0” key presses in trials without a deviant) were removed from the main trials and the false alarm rate was determined for each participant.

**Figure 1.**
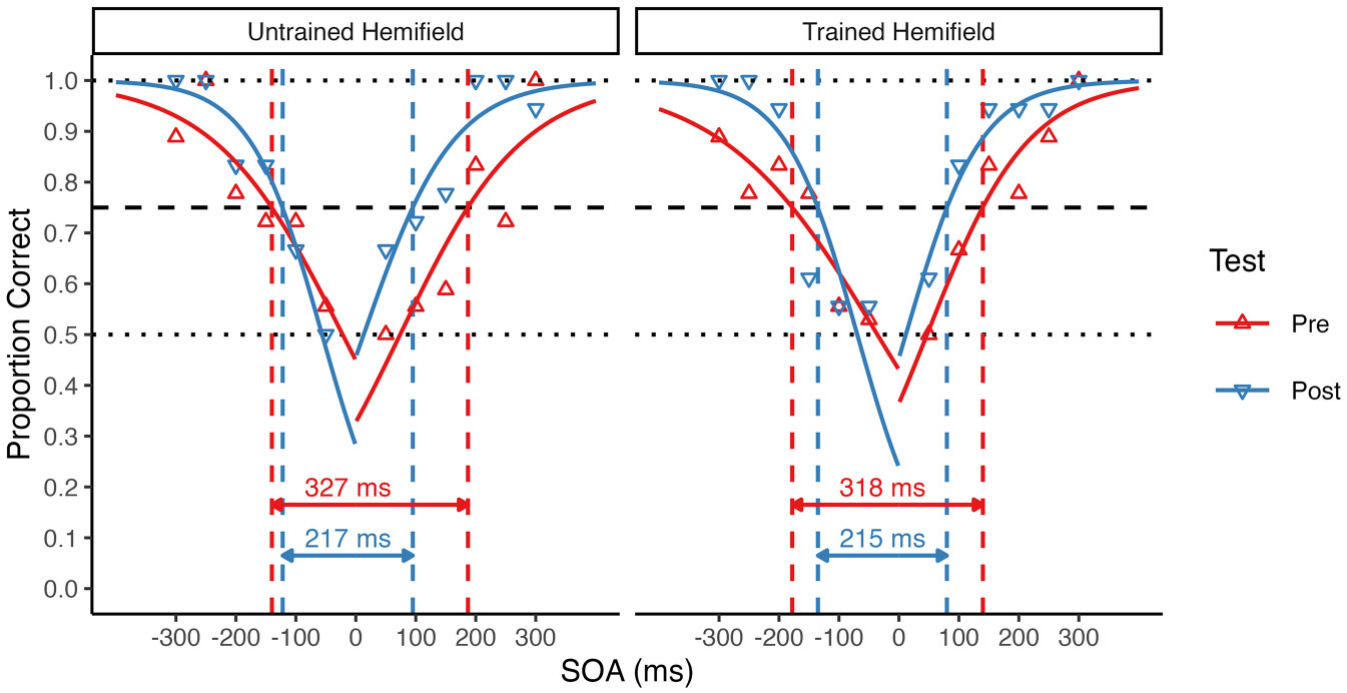
Estimation of individual TBW size. Triangles show the proportion of correct responses at each SOA for a single participant. Data are shown separately for the pretest (*upward triangles*) and posttest (*downward triangles*) in the untrained (*left panel*) and trained (*right panel*) hemifield. For each condition, accuracy values were fitted by two logistic regressions, one for negative (auditory leading) and one for positive (visual leading) SOAs. TBW size was derived from the fitted psychometric curves at half the difference between an individual's lowest accuracy point in the baseline session (here 0.5, lower dotted line) and perfect performance (1.0, upper dotted line), indicated by the dashed horizontal line (here at 0.75). The total TBW size for this participant decreased from pretest to posttest by 100 ms in the untrained hemifield and by 103 ms in the trained hemifield (see dashed vertical lines).

*Note*. SOA = stimulus onset asynchronies; TBW = temporal binding window.

The remaining main trials were then used to estimate the TBW for each participant and condition following the procedure described in Powers et al. (2009, 2012). For this purpose, accuracy (i.e., proportion of correct responses) was determined for each SOA and the resulting values were fitted by two logistic regressions, one for negative (auditory leading) SOA values, and one for positive (visual leading) SOA values (excluding the 0 ms SOA condition for which correctness of the response is undefined). The size of the TBW was then determined separately for the left (auditory leading) and the right (visual leading) side as the SOA at which the respective psychometric curve reached half the difference between an individual's lowest accuracy value in the baseline session and perfect performance (see Figure 1). Next, the total TBW size was calculated as the sum of the left and right TBW size. Changes in TBW size from pre- to post-test were then compared between the trained and the untrained hemifield with a repeated-measures ANOVA with factors of test (pretest vs. posttest) and Hemifield (untrained vs. trained). We additionally analyzed changes in accuracy (i.e., mean proportion of correct responses). All statistical tests were additionally performed as Bayesian hypothesis tests using standard priors in JASP version 0.18.1 (Wagenmakers et al., 2018), and Bayes Factors (BF_incl_ for ANOVA main effects and interactions) are reported to indicate the evidential value for the null or alternative hypothesis, respectively.

## Results

### Deviant and Catch Trial Performance

The hit rate in deviant trials, which required responding to a flickering of the central fixation LED, was overall high (see Table 1) and significantly improved from pretest (*M* = 85.6%, *SEM* = 2.7) to posttest (*M* = 91.8%, *SEM* = 2.7), *F*(1, 32) = 6.48, *p* = .016, η^2^_G_ = .04, BF_incl_ = 41.97. Performance in deviant trials did not differ between the untrained and trained hemifield as there was neither a significant main effect of Hemifield, *F* < 1, BF_incl_ = 0.16, nor a significant interaction of Test and Hemifield, *F* < 1, BF_incl_ = 0.18. Compared to the test phases, hit rates in deviant trials were similar during the training phase in which feedback was provided (*M* = 90.0%, *SEM* = 1.9).

**Table 1.** Mean pretest and posttest performance (with SEMs) in the untrained and trained hemifield.

	Untrained hemifield		Trained hemifield	
	Pretest	Posttest	Pretest	Posttest
Deviant hit rate	86.1% (3.1)	91.4% (2.4)	85.1% (2.9)	92.3% (2.8)
Catch trial bias	1.69 (0.03)	1.76 (0.03)	1.68 (0.03)	1.75 (0.03)
SJ accuracy (all SOAs)	79.6% (1.3)	82.3% (1.2)	78.8% (1.2)	82.6% (1.1)
SJ accuracy (negative SOAs)	78.6% (1.6)	79.9% (1.8)	77.8% (1.6)	80.0% (1.6)
SJ accuracy (positive SOAs)	80.6% (1.3)	84.7% (1.2)	79.8% (1.4)	85.2% (1.3)
TBW size (total)	221 ms (14)	193 ms (12)	234 ms (15)	195 ms (13)
TBW size (left)	115 ms (11)	113 ms (10)	127 ms (11)	115 ms (11)
TBW size (right)	106 ms (6)	80 ms (7)	107 ms (9)	80 ms (7)

*Note.* Catch trial bias refers to mean response (between 1 and 2) in trials in which both audiovisual events were synchronous; unbiased responses (i.e., 50% first interval and 50% second interval) would result in a value of 1.5. SJ = simultaneity judgment. TBW = temporal binding window; SOA = stimulus onset asynchronies.

False alarms (i.e., deviant responses in main trials) were rare and occurred in less than 1% of trials overall, with no significant differences between tests and hemifields (all *p* ≥ .078, BF_incl_ ≤ 0.62).

In line with previous reports (Powers et al., 2009), responses in catch trials with two synchronous (instead of one synchronous and one asynchronous) audiovisual stimuli indicated a bias toward perceiving the synchronous audiovisual stimulus in the second interval (see Table 1). This bias tended to increase from pretest (*M* = 1.69, *SEM* = 0.03) to posttest (*M* = 1.76, *SEM* = 0.02), *F*(1, 32) = 3.78, *p* = .061, η^2^_G_ = .05, BF_incl_ = 3.40. The bias did not differ between the untrained and trained hemifield as there was no significant main effect of Hemifield, *F* < 1, BF_incl_ = 0.15, and no significant interaction of Test and Hemifield, *F* < 1, BF_incl_ = 0.13.

### SJ Accuracy

**Figure 2.**
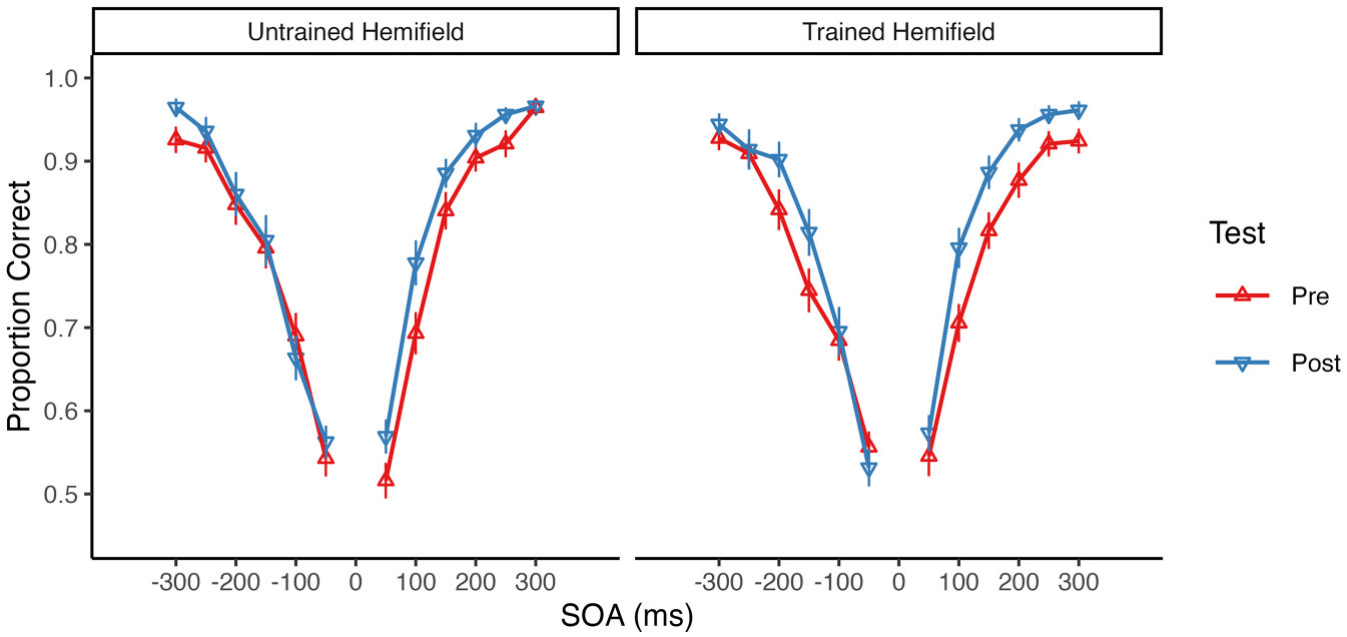
Group mean accuracy at each stimulus onset asynchrony (SOA) in the untrained and trained hemifield. The mean proportion of correct responses is shown separately for the pretest (*upward triangles*) and the posttest (*downward triangles*). Negative SOA values indicate auditory leading and positive SOA values indicate visual leading trials. Error bars indicate the *SEM*.

**Figure 3.**
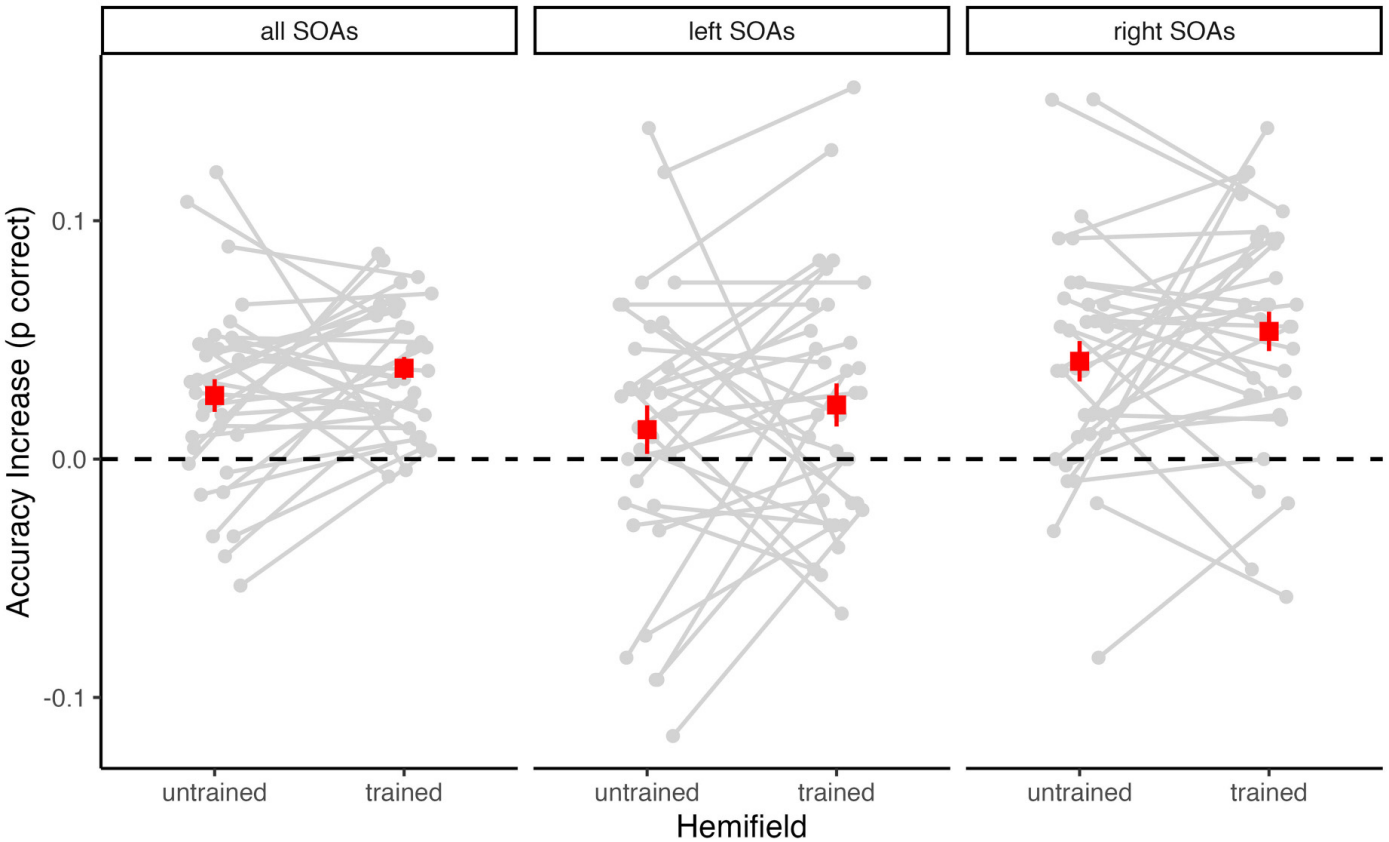
Changes in accuracy from pretest to posttest for each participant. The difference in the proportion of correct responses (posttest minus pretest) is shown separately for the untrained and the trained hemifield. Positive values indicate performance increases and negative values indicate performance decreases. Superimposed squares indicate the group mean values (with *SEM*). The left panel shows changes in accuracy across all stimulus onset asynchrony (SOA) values. Additionally, accuracy changes were calculated separately for the left side (middle panel) of the SOA distribution (i.e., negative or auditory leading SOAs) and the right side (right panel) of the SOA distribution (i.e., positive or visual leading SOAs).

The mean pretest and posttest accuracy rates for each SOA are shown in Figure 2 separately for the untrained and trained hemifields. Overall, accuracy increased from pre- to post-test in both hemifields. Accordingly, a 2 × 2 repeated-measures ANOVA with factors of test (pretest vs. posttest) and Hemifield (untrained vs. trained) revealed a highly significant main effect of Test, *F*(1, 32) = 53.37, *p* < .001, η^2^_G_ = .05, BF_incl_ = 246,687.12. There was, however, no significant main effect or interaction involving Hemifield (both *p* ≥ .152, BF_incl_ ≤ 0.65), suggesting that SJ accuracy equally increased in both the untrained and the trained hemifield (see Figure 3).

Descriptively, the increase in accuracy from pretest to posttest was more pronounced for positive (i.e., visual leading) SOAs than for negative (i.e., auditory leading) SOAs (see Table 1). Moreover, across tests and hemifields, accuracy was generally higher for positive SOAs (*M* = 82.5%) than for negative SOAs (*M* = 79.2%). Therefore, we additionally computed separate ANOVAs for negative and positive SOAs. These analyses revealed a highly significant main effect of Test for positive SOAs, *F*(1, 32) = 46.60, *p* < .001, η^2^_G_ = .09, BF_incl_ = 148,271.40, and a weaker but still significant main effect of Test for negative SOAs, *F*(1, 32) = 5.03, *p* = .032, η^2^_G_ = .01, BF_incl_ = 1.40. There was no significant main effect of Hemifield and no significant Test×Hemifield interaction, neither for positive SOAs (both *p* ≥ .192, BF_incl_ ≤ 0.38) nor for negative SOAs (both *p* ≥ .363, BF_incl_ ≤ 0.22). Thus, for both negative and positive SOAs, there was no significant difference in accuracy increase between the untrained and the trained hemifield (see Figure 3).

### Temporal Binding Window

**Figure 4.**
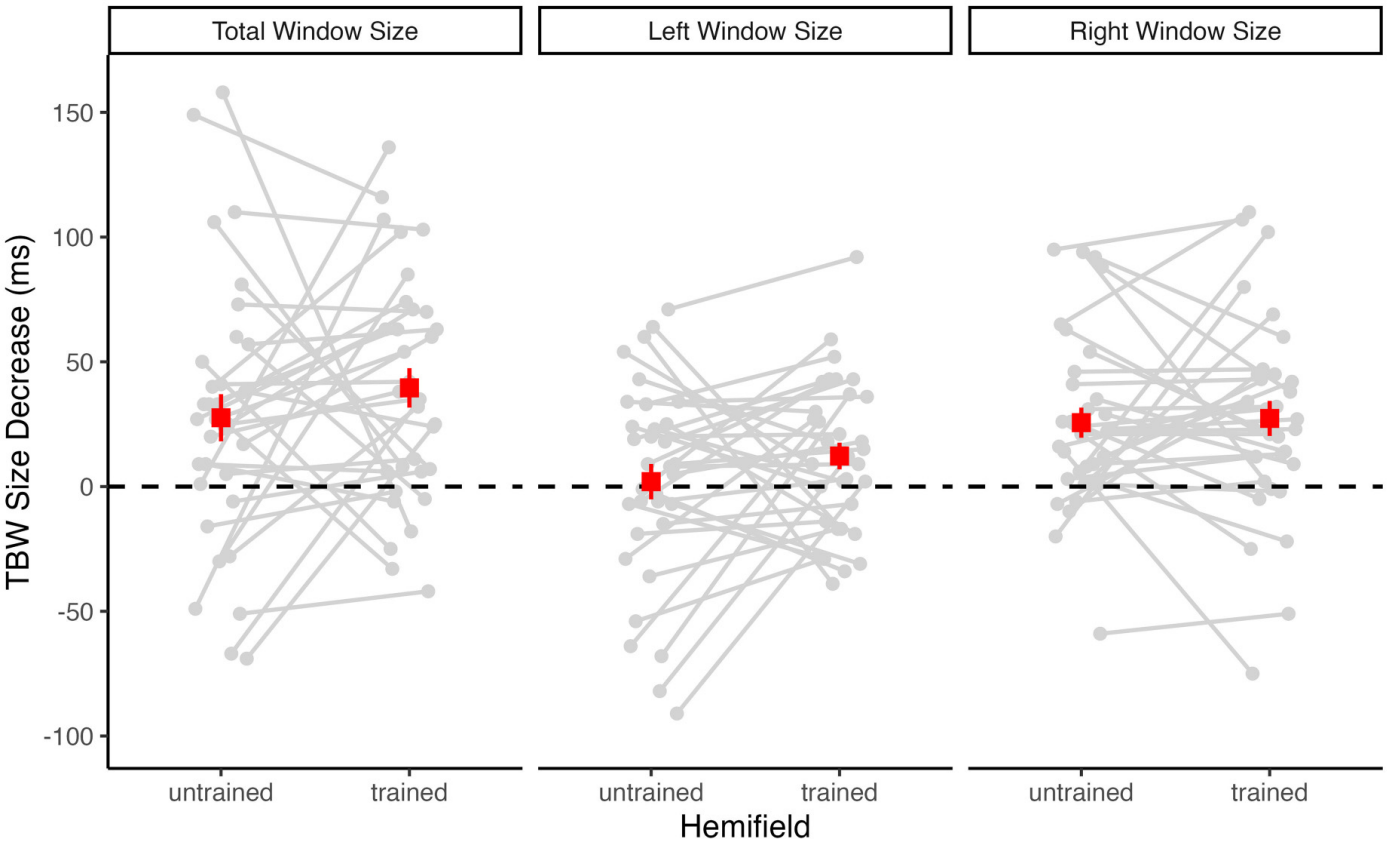
Changes in TBW size from pretest to posttest for each participant. The difference in the TBW size (pretest minus posttest) is shown separately for the untrained and the trained hemifield. Positive values indicate TBW size decreases and negative values indicate TBW size increases. Superimposed squares indicate the group mean values (with *SEM*). The left panel shows TBW changes for the total window size (left plus right window size). Additionally, TBW changes were calculated separately for the left window (middle panel) covering the negative (auditory leading) SOAs and the right window (right panel) covering the positive (visual leading) SOAs.

*Note*. SOA = stimulus onset asynchronies; TBW = temporal binding window.

Because accuracy rates are only an indirect measure of TBW size, we additionally determined pretest and posttest TBWs for the untrained and trained hemifields for each participant from two psychometric curves fitted to the individual accuracy rates, one for negative (auditory leading) and one for positive (visual leading) SOA values (see Figure 1). For the participant shown in Figure 1, the total TBW size (i.e., the sum of the left and right TBW size) decreased from 327 to 217 ms in the untrained hemifield, and from 318 to 215 ms in the trained hemifield. Across participants, the mean total TBW decrease was of similar size in the untrained (*M* = 28 ms) and trained (*M* = 40 ms) hemifield. Accordingly, and consistent with the accuracy rate analysis reported above, a 2 × 2 repeated-measures ANOVA with factors of test (pretest vs. posttest) and Hemifield (untrained vs. trained) revealed a highly significant main effect of Test, *F*(1, 32) = 27.74, *p* < .001, η^2^_G_ = .04, BF_incl_ = 791.27. There was no significant main effect or interaction involving Hemifield (both *p* ≥ .308, BF_incl_ ≤ 0.43), suggesting that TBW size equally decreased in both the untrained and the trained hemifield (see Figure 4).

Descriptively, the decrease in TBW size from pretest to posttest was more pronounced for the right (visual leading) window side than for the left (auditory leading) window side (see Figure 4 and Table 1). Moreover, across tests and hemifields, right TBWs were generally smaller (*M* = 93 ms) than left TBWs (*M* = 101 ms). Therefore, we additionally computed separate ANOVAs for the left and the right window side. These analyses revealed that the main effect of Test was highly significant for the right window side, *F*(1, 32) = 24.62, *p* < .001, η^2^_G_ = .10, BF_incl_ = 741.10, but not significant for the left window side, *F*(1, 32) = 2.22, *p* = .146, η^2^_G_ < .01, BF_incl_ = 0.45. There was no significant main effect of Hemifield and no significant Test×Hemifield interaction, neither for the right window side (both *p* ≥ .826, BF_incl_ ≤ 0.21) nor for the left window side (both *p* ≥ .134, BF_incl_ ≤ 0.41). Thus, for both window sides, there was no significant difference in TBW size decrease between the untrained and the trained hemifield (see Figure 4).

## Discussion

In the present study, we tested the location-specificity of perceptual learning effects in multisensory temporal processing. To this end, participants received feedback about their performance in an audiovisual SJ training phase in which stimuli were exclusively presented in either the left or the right hemifield. Before and after the training phase, the size of the TBW was assessed for both the trained and the untrained hemifield. Three main findings emerged from our study. First, consistent with previous reports (O’Brien et al., 2020; Powers et al., 2009, 2012; Theves et al., 2020; Zerr et al., 2019), SJ training resulted in a significant decrease of the TBW size in the posttest on the following day. Thus, our results further substantiate that a short perceptual training with feedback is sufficient for improving audiovisual temporal discrimination abilities in adult individuals (for a recent review, see O’Brien et al., 2023). Second, post-training performance improvements were more pronounced for visual-leading SOAs than for auditory-leading SOAs. This finding is consistent with previous studies (Cecere et al., 2016; Powers et al., 2009) and most likely reflects the high prevalence of visual leading stimuli with varying SOAs based on distance in the natural environment, which might result in a higher adaptivity of temporal representations for visual leading as compared to auditory leading stimuli. Third, and most importantly, performance improvements were indistinguishable between stimuli that were presented in the trained hemifield and stimuli that were presented in the untrained hemifield. This result indicates that perceptual training effects on audiovisual temporal binding completely generalized across spatial locations in our study, suggesting that learning had occurred at location-independent processing stages.

We did not obtain any evidence for a reduced perceptual learning effect in the untrained compared to the trained hemifield despite sufficient statistical power of our sample size. In fact, complementary Bayesian hypothesis tests consistently yielded evidence in favor of the null hypothesis (i.e., BFs < 1) of equal learning effects in both hemifields (Wagenmakers et al., 2018). This conclusion, however, critically depends on the assumption that our experimental manipulation had indeed successfully confined stimulus presentations to the left or right hemifield, respectively. While the constrained head position in our experiment factually ensured that left and right stimulus locations differed in head-centered (auditory) spatial coordinates, systematic eye movements (e.g., fixation of the audiovisual stimuli instead of the central fixation LED) could theoretically have resulted in similar eye-centered (visual) spatial coordinates for the two stimulus locations, thereby potentially foiling the location-specificity of the training procedure. As in previous studies that had investigated the spatial specificity of crossmodal temporal recalibration (Heron et al., 2012; Ho et al., 2015; Ju et al., 2019; Yuan et al., 2012), we did not directly measure eye position with an eye tracker and, therefore, cannot entirely exclude this possibility. However, to ensure that participants followed the task instructions and maintained fixation throughout the test and training phases, the experimental procedure included a demanding visual deviant detection task at central fixation. Performance in the central deviant detection task was high but below ceiling with a mean hit rate around 90%, suggesting that participants had, as instructed, maintained central fixation throughout the experiment. Thus, it appears unlikely that the spatial generalization of the crossmodal temporal learning effect seen in our study was due to systematic eye fixation shifts across participants. Moreover, it appears unlikely that crossmodal temporal learning effects would have occurred in an exclusively eye-centered reference frame. Systematic manipulations of eye-centered relative to head-centered spatial coordinates in audiovisual spatial recalibration studies have shown that recalibration most likely emerged in either a hybrid or head-centered reference frame (Lokša & Kopčo, 2023; Kopčo et al., 2009; Watson et al., 2021). Thus, even in the presence of systematic eye fixation shifts, the difference in head-centered coordinates of our stimuli would likely have allowed for spatially specific learning effects to emerge, although the role of eye movements remains to be directly tested by using eye-tracking in future studies.

Another possibility to consider are unspecific test repetition effects, which could have resulted in an increased SJ performance in the untrained hemifield independently from the unilateral training phase. Future studies might consider including a dedicated control group to account for this possibility. It is, however, well-established that performance in crossmodal temporal binding tasks is highly stable across multiple measurements (Basu Mallick et al., 2015; Odegaard & Shams, 2016). Concordantly, previous studies have consistently shown that training-induced improvements in audiovisual SJ performance depended on the availability of feedback during training and did not occur in control conditions with simple passive exposure to the training stimuli (Huang et al., 2022; Powers et al., 2009; Stevenson et al., 2013; Theves et al., 2020). In other studies, training effects were selectively abolished for easy (versus hard) task difficulty during training (De Niear et al., 2016), for low (versus high) visual intensity stimuli during training (Horsfall et al., 2021), or for unimodal (versus crossmodal) training stimuli (Zerr et al., 2019), corroborating that performance improvements are dependent on a specific perceptual training protocol rather than reflecting unspecific test repetition effects. In light of this unequivocal evidence and in accord with previous research (McGovern et al., 2022; O’Brien et al., 2020; Powers et al., 2012), we did not consider the inclusion of a dedicated control group with passive exposure to the crossmodal stimuli necessary for interpreting crossmodal temporal training effects. In addition, we used a stringent 2IFC test procedure in our study which excludes the possibility that simple changes in response criterion, rather than perceptual learning, could account for the improved post-training performance (Powers et al., 2009; Stevenson & Wallace, 2013). Taken together, this strongly suggests that performance improvements in our study were genuinely induced by the feedback training phase, and hence generalized from trained to untrained spatial locations.

Neuroimaging studies of audiovisual temporal processing have identified a distributed network of brain regions including unisensory auditory and visual cortices as well as multisensory superior temporal cortex (Noesselt et al., 2007; Powers et al., 2012; Stevenson et al., 2010) and fronto-parietal areas (Adhikari et al., 2013; Binder, 2015; Lu et al., 2014) which underlies the detection of audiovisual simultaneity and asynchrony. Improvements in SJ performance following feedback training were found to be associated with increased functional connectivity within this network (Powers et al., 2012) as well as with elevated beta-band activity in electrophysiological recordings (Theves et al., 2020). The latter finding suggests that the improved temporal acuity after training might be due to an enhanced top-down modulation of sensory processing which has been linked to beta oscillations (Keil & Senkowski, 2018; Senkowski & Engel, 2024). If audiovisual perceptual learning indeed primarily emanated from higher-level areas rather than from lower-level sensory areas which are sensitive to both temporal and spatial crossmodal disparities (Bonath et al., 2014), a generalization of temporal learning to untrained spatial locations would be expected in line with the present results.

Our findings do, however, not necessarily imply that spatial generalization is a general feature of all crossmodal perceptual learning protocols in the temporal domain. The reverse hierarchy theory of perceptual learning (Ahissar & Hochstein, 2004) posits that perceptual learning is a top-down guided process which may progress from higher- to lower-level areas if the task requires fine-grained discriminations that cannot be resolved within the initially involved higher-level cortical representations. The involvement of lower-level sensory representations would then entail a stronger learning specificity (e.g., for the trained spatial location). Similar ideas were put forward for spatial attention effects. For example, the precision of spatial attention effects was found to depend on the visual resolution that was needed for task performance (Bartsch et al., 2023). Concordant results were observed in a crossmodal temporal recalibration task: When visuotactile stimuli with a consistent temporal asynchrony were exclusively presented in one of the two hemifields, subsequent recalibration of the PSS generalized to the untrained hemifield (Ho et al., 2015), similar to the spatial generalization of feedback-induced perceptual training effects seen in our study. However, concurrent adaptation to opposite asynchronies in the left and right hemifield resulted in hemifield-specific PSS shifts in opposite directions (Heron et al., 2012; Ho et al., 2015), suggesting that temporal recalibration can become spatially specific if the crossmodal temporal conflict cannot be resolved at a higher (location-independent) level.

Future studies will need to determine if and under which conditions feedback-based perceptual training would result in location-specific improvements in crossmodal temporal acuity. Parameters that might modulate learning specificity include the duration of the training phase as well as the difficulty of the training task. In line with the reverse hierarchy theory (Ahissar & Hochstein, 2004) and findings in crossmodal recalibration tasks (Bruns & Röder, 2015, 2019a; Ju et al., 2019), learning specificity might be expected to increase with longer training durations. In the present study, the training phase was relatively short (a single session of 360 training trials), which might have prevented spatially specific training effects. However, the training duration in our study was selected based on previous behavioral studies which had shown that perceptual learning effects in the SJ task were already maximal after only one training session and did not increase further with additional training (Powers et al., 2009; Zerr et al., 2019). Moreover, in crossmodal temporal recalibration studies, spatial specificity has been observed after a single audiovisual exposure session (Heron et al., 2012; Yarrow et al., 2011; Yuan et al., 2012), suggesting that the amount of training in the present study would likely have been sufficient to induce similar effects if spatial specificity existed in feedback-based audiovisual temporal learning and depended on similar underlying mechanisms as in temporal recalibration.

It is less clear how task difficulty would affect the spatial generalization of the training effects. Studies of unisensory visual perceptual learning have consistently reported that more difficult training conditions result in a greater location-specificity of the training effects (Ahissar & Hochstein, 2004; Hung & Seitz, 2014). The effects of task difficulty on the location-specificity of crossmodal temporal training effects has not been assessed yet. However, several recent studies have investigated the transfer of perceptual training effects in the SJ task to other multisensory tasks including the sound-induced flash illusion, the spatial ventriloquism illusion, and the redundant target effect (McGovern et al., 2016, 2022; O’Brien et al., 2020; Powers et al., 2016; Setti et al., 2014; Sürig et al., 2018; Zerr et al., 2019). The emerging picture from these studies is that generalization to new multisensory tasks only occurred in difficult training conditions in which participants were constantly trained at their individual threshold (O’Brien et al., 2023). For example, Sürig et al. (2018) directly compared an experimental group that was trained with an adaptive staircase procedure and a control group in which SOAs were presented randomly. They found that adaptive training resulted in faster improvements in the SJ task as well as in transfer to other multisensory tasks which both were not seen in the control group. Whether and how more difficult training conditions would affect spatial generalization in the SJ task remains to be determined. However, perceptual training in the SJ task was found to be only effective if the training phase focused on the most difficult (shortest) SOAs as training effects were abolished after trainings including mainly medium-difficult or easy SOAs (De Niear et al., 2016). Accordingly, we used only the most challenging SOAs from the baseline test in the training phase and successfully replicated temporal learning effects with this procedure, suggesting that our training condition was sufficiently difficult.

Taken together, our results extend previous reports of feedback training-induced improvements in audiovisual temporal acuity by showing that these training effects generalize across spatial locations. This finding suggests that perceptual learning in the temporal domain primarily involved higher, location-independent processing stages. Future studies could consider varying training durations and intensities to determine if spatial specificity of the training effects might emerge at a later stage of learning, as well as including eye-tracking to clarify whether spatial specificity becomes more likely within a stable eye-centered reference frame. Ultimately, knowledge about the conditions under which crossmodal temporal learning generalizes and their underlying mechanisms might contribute to the development of clinical interventions targeted at the enlarged TBW associated with neuropsychiatric disorders such as autism and schizophrenia as well as with increased susceptibility to falls in the elderly (O’Brien et al., 2023; Wallace et al., 2020; Zhou et al., 2020).
